# Description of *Callophyllamacrocephala* sp. n. from southern Tanzania

**DOI:** 10.3897/zookeys.818.32269

**Published:** 2019-01-23

**Authors:** Renzo Perissinotto

**Affiliations:** 1 School of Environmental Sciences, Nelson Mandela University, P.O. Box 77000, Port Elizabeth 6031, South Africa Nelson Mandela University Port Elizabeth South Africa

**Keywords:** Afrotropical region, *
Callophylla
*, Cetoniinae, new species, Tanzania

## Abstract

A male cetoniine specimen from the old Schürhoff collection currently deposited in the Ditsong National Museum of Natural History (Pretoria, South Africa), was recently submitted for identification and has been found to represent a yet undescribed species of the poorly-known genus *Callophylla* Moser, 1916. The species is named *C.macrocephala***sp. n.**, in recognition of its wider than average head, particularly at the level of the clypeus, and originates from the southern Tanzanian highlands, near the Tanzania-Zambia border town of Nakonde. This brings the total number of species now known for this genus to four, two from West Africa and two from East-Central Africa. All species were described on the basis of a male only, or this and a few extra specimens. The female is only known for the two West African species, *C.costata* Moser, 1916 and *C.lamottei* Antoine, 2007. A dichotomous key for the identification of the species of this genus is presented for the first time. It is suggested that the genus may be constituted of high altitude specialists, with a short period of activity and no ability to feed at the adult stage.

## Introduction

The genus *Callophylla* Moser, 1916 was described along with the type species *C.costata* Moser, 1916, based on a male from central Cameroon. What was at first believed to be the female of the same species was described much later by Ruter (1954), on the basis of a specimen collected on Mount Nimba in Guinea. It was only in 2007 that sufficient material had finally become available to allow Antoine (2007) to describe the correct female of *C.costata* along with a new species, *C.lamottei* Antoine, 2007, to which the female described earlier by Ruter (1954) was recognised to belong. Further to this, a third species, *C.takanoi* Legrand, 2015 was recently described on the basis of a single male collected in western Zambia during a survey undertaken to the region by the British Natural History Museum. Thus, so far only a handful of specimens are known for the entire genus and of these apparently only two are female, one belonging to *C.costata* and the second to *C.lamottei*. The female of *C.takanoi* is undescribed and information on habitat and distribution of the three species of the genus is extremely limited.

A new species, recently recognised from an old specimen originating from the “Deutsch-Ostafrika” collection of Schürhoff and currently preserved in the Ditsong National Museum of Natural History (formerly Transvaal Museum, Pretoria, South Africa), is here described, thus providing additional, valuable information on the diversity of this poorly known genus.

## Materials and methods

The holotype and only known specimen for this new species was submitted by Ms Ruth Müller of the Ditsong National Museum of Natural History (Pretoria, South Africa) for identification, as part of a loan to the author of several cetoniine specimens which are currently under review. The usual terminology of Krikken (1984) and Holm and Marais (1992) is followed in this study for the description of morphological characters. Specimen total length and maximum width were measured using a Vernier calliper, from the anterior margin of the clypeus to the apex of the pygidium and at the widest point of the elytra, respectively.

Photos of whole specimen dorsal and ventral habitus were taken with a Nikon CoolPix S9700 digital camera with macro setting, while photos of the male genitalia and other anatomical details were obtained using a Nikon DigitalSight DS-Fi2 camera attached to a Nikon SMZ25 dissecting microscope. The background was removed from the photos using Microsoft Word 2010 (Picture Tools), in order to increase clarity of resolution. The Combine ZP Image Stacking Software by Alan Hadley (alan@micropics.org.uk) was used to obtain z-stacking composite images.

Data on distribution and period of adult activity of the various species of the genus *Callophylla* were obtained from Moser (1916), Ruter (1954), Sakai and Nagai 1998, Antoine (2007), Legrand (2015), Beinhundner (2017) and from holders and curators of relevant collections (as per Acknowledgements section).

## Taxonomy

### Callophylla macrocephala sp. n.

Taxon classificationAnimaliaColeopteraCetoniidae

http://zoobank.org/8DE7C0D2-AEC8-4351-8841-D69D44A8D9BF

Figures 1 , 2

#### Diagnosis.

This species can easily be separated from all the other species currently known in the genus by its remarkably wide clypeus, the brightness of its body surface and the scattered round to horseshoe punctures on the pronotum (dense and rugose in all the other species). Of the four species currently known in the genus *Callophylla*, *C.macrocephala* appears to be most closely related to *C.takanoi*, which occurs in Zambia (Ikelenge), but on its northwestern corner at the border with the DRC and Angola. Conversely, the only record currently known for *C.macrocephala* (“Nakonde Hochland”) is from the border region between southern Tanzania and north-eastern Zambia.

The two species can easily be separated on the basis of their key differences at the level of the clypeal width, pronotal tubercle and sculpture, aedeagal parameres and the general body colour and ornamentation. In particular, the clypeus of *C.macrocephala* is as wide as the total length of its head (from the tip of the clypeus to the anterior margin of the pronotum), while in *C.takanoi* it is shorter by about 30%. The pronotum of *C.macrocephala* exhibits a very prominent tubercle on its mid anterior margin, while this is absent in *C.takanoi*. Additionally, the pronotum of *C.macrocepala* is completely black and characterized by scattered, round to horseshoe punctures, while in *C.takanoi* it is brickred on the sides and black at middle, with dense rugose sculpture throughout the surface. Finally, the parameres of *C.macrocephala* are much longer and narrower than those of *C.takanoi* and also with very few, short setae at the apex.

#### Description of holotype.

**Figure 1.**
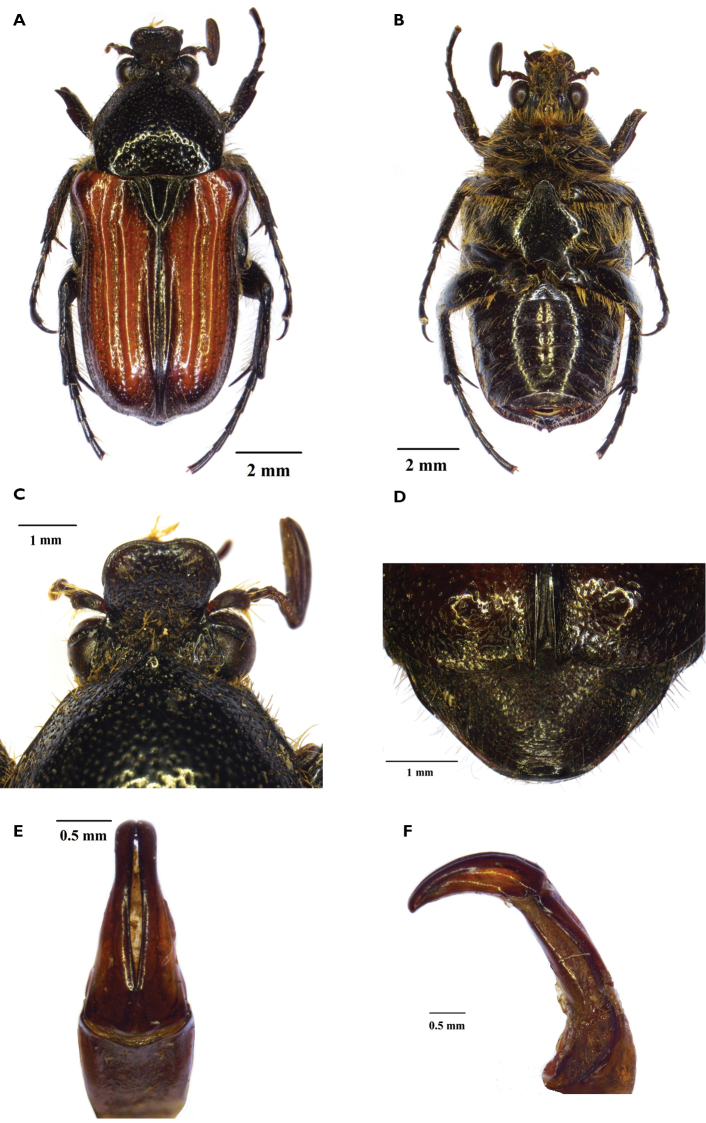
Dorsal (**A**) and ventral (**B**) habitus of *Callophyllamacrocephala* sp. n., with details of clypeus (**C**), pygidium (**D**) and aedeagus in dorsal (**E**) and lateral (**F**) view (photographs Lynette Clennell).

**Male.** (Fig. 1A–F) *Size*. Length 12.8 mm; width 5.3 mm.

*Body*: Shiny and elongate, black to light brown and ochreous in colour; with remarkable punctuation and long but scattered setae through most of dorsal surface (Fig. 1A).

*Head*. Wide and completely black; clypeus deeply concave and sharply upturned at anterior margin, sinuate at centre (Fig. 1C); entire surface covered in round to horseshoe

punctures, with exception of ocular canthus, with tawny-coloured setae emerging at centre of each puncture and becoming particularly long towards vertex (Fig. 1C); antennal club dark brown and black, slightly longer than flagellum; flagellum dark brown; pedicel dark brown with lighter head attachment and bearing clusters of long, erected yellowish setae.

*Pronotum*. Surface entirely black and shiny with numerous but well-spaced punctures; punctures round on disc becoming horseshoe towards pre-scutellar arch; shape heptagonal and remarkably elevated at anterior margin, forming prominent tubercle at centre; antero-lateral margins carinate, postero-lateral smooth; posterior margin slightly sinuate with pre-scutellar arch smooth; medium to long yellowish setae scattered throughout lateral declivity and margins (Fig. 1A).

*Scutellum.* Completely black and shiny; smooth on disc and exhibiting only minor geminate striae on antero-lateral margin; narrowly triangular with lateral margins much longer than base and sharp apex; lateral grooves exceptionally deep and wide (Fig. 1A).

*Elytron*. Shiny throughout; ochreous on disc, but dark brown to black on all margins except behind pronotal extra-scutellar area; costae very pronounced and typical of members of the genus; sub-humeral arch very deep, but both humeral and apical calluses poorly developed; paired horseshoe punctures lining entire surface of intercostal area, with long and erect tawny-coloured setae emerging at centre of most punctures; apical margin smoothly rounded, with a moderately-developed proximal spine; apical and postero-lateral declivities remarkably steep (Fig. 1A).

*Pygidium*. Closer to semicircular than triangular in shape and slightly convex; Dark brown to black and covered in dense rugose sculpture; short to medium yellow setae scattered around the disc, becoming longer and more numerous on apico-lateral margins.

*Legs*. Slender and elongate, with apical tarsal segments approximately twice as long as preceding ones; protibia bidentate, with second tooth blunt and poorly developed, with fine longitudinal ridges, coarse horseshoe punctures and short yellow setae, becoming longer and denser on inner margin; meso- and metatibia with longer and denser yellow setae, with striolate surfaces and mid spine on outer carina moderately developed; spurs long and acuminate, approximately twice as long in metatibia than in mesotibia (Fig. 1A, B).

*Ventral surface*. Completely black and shiny; with small and scattered horseshoe to round punctures throughout surface, except on mesometasternal lobe and on central area of abdominal sternites; pubescence long and dense, but shorter and scattered on abdomen and absent on mesometasternal lobe; mesosternal lobe smoothly rounded and slightly projecting anteriorly; abdominal sternites with visible concavity and groove at centre; metacoxa with remarkable carina separating ventral from lateral portion.

*Aedeagus*. Parameres elongate and slender, with apex smoothly rounded and bearing few scattered setae at centre (Fig. 1C); dorsal lobes of same width of ventral lobes and perfectly parallel throughout length (Fig. 1C).

*Derivatio nominis.* The name of this species reflects its particularly wide head, in comparison to that of all other known congeneric species.

**Female.** The female of this species, like that of its closest relative *C.takanoi*, is unknown, but is expected to exhibit a remarkable dimorphism, with broad characteristics similar to those previously described for *C.costata* and *C.lamottei* from West Africa (Antoine 2007). In those species, the main differences lie in the female exhibiting a tridentate and substantially enlarged protibia, in comparison to the male. The antennal clubs are almost twice as long in the male, while the general body shape is generally broader and more globose in the female. Additionally, typically the female meso- and metatibial teeth are more pronounced than those of the male.

#### Distribution.

The only known specimen of *C.macrocephala* was collected in the “Nakonde Hochland” area of the old “Deutsch-Ostafrika”. This colony included the present day mainland part of Tanzania and although the town of Nakonde falls within Zambia, the highlands area formed part of the Lindi District of the old German colony (Schnee 1920, Vol II, p. 457). Thus, the type locality is obviously just across the Zambian border town of Nakonde. It is most likely though that the distribution range of this species extends to the nearby mountainous regions of both Zambia and Malawi.

#### Remarks.

It may be of interest to note that despite the specimen carrying an unequivocal label (“Sammlung Schürhoff”), that identifies it as having belonged to the collection of this prolific entomologist of the early 20^th^ century, no reference to it could be found in his extensive series of publications on the Cetoniinae of the World (“Beiträge zur kenntnis der Cetoniden”).

#### Type material.

Holotype (♂): Tanzania (“D. O. Afr”), Nakonde Hockland, Sammlung Schürhoff (TMSA “F”, “7”).

**Figure 2.**
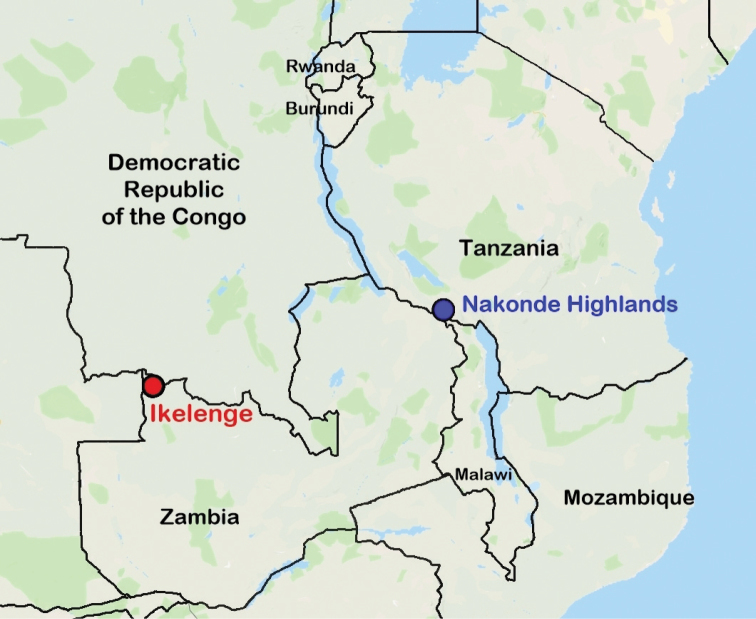
Type localities of *Callophyllamacrocephala* sp. n. (blue circle) and *Callophyllatakanoi* Legrand, 2015 (red circle) in Central – East Africa.

### Identification key to the species of the genus *Callophylla*

**Table:** 

1	Internal elytral costae fully developed and raised, external costae poorly raised to obsolete; body shape moderately elongate; West African distribution	**2**
–	Internal and external elytral costae equally well-developed and raised; body shape remarkably elongate; Central to East African distribution	**3**
2	Anterior margin of clypeus straight in male and weakly sinuate in female; antero-lateral angles of pronotum situated anteriad of mid pronotal length; lateral margin of metacoxae carinate; recorded from Cameroon, Gabon, Congo and Democratic Republic of Congo	***C.costata* Moser, 1916**
–	Anterior margin of clypeus indented in both sexes; antero-lateral angles of pronotum situated at middle of pronotal length; lateral margin of metacoxae smoothly rounded; recorded from Guinea and Ivory Coast	***C.lamottei* Antoine, 2007**
3	Body surface partly shiny; clypeal width shorter than total head length; pronotum without tubercle on anterior margin; recorded from Zambia (Ikelenge)	***C.takanoi* Legrand, 2015**
–	Body surface completely shiny; clypeal width as long as total head length; pronotum with prominent tubercle on anterior margin; recorded from Tanzania (Nakonde Highlands)	***C.macrocephala* sp. n.**

## Discussion

*Callophyllamacrocephala* sp. n. represents the fourth species described within a very unique and poorly known genus. Very few specimens are known for this genus, and most are from Cameroon and belong to the species *C.costata*. The female of *C.takanoi* and *C.macrocephala* remains unknown, while only one female of each *C.costata* and *C.lamottei* are known with certainty (Ruter 1954, Antoine 2007, Beinhundner 2017).

A remarkable colour variation has been observed in the dorsal habitus of *C.costata*, with specimens ranging from completely reddish-ochre to dark brown and even completely black. Most specimens, however, exhibit a combinations of the lighter colours with a variable degree of black ornamentation on pronotum and elytra (Moser 1916, Beinhundner 2017). The head and scutellum appear to show the most conservative trend, in that they are always predominantly black, with few outstanding exceptions. The antennal clubs are invariably reddish to brown, even in the darkest specimens (Beinhundner 2017). Unfortunately, no similar conclusions can be drawn for the other three species, due to lack of material beyond the holotypes. However, in *C.lamottei* the male paratype is completely black, while the female holotype is reddish-brown with black areas across part of the dorsal surface (Antoine 2007). It seems likely, therefore, that a wide variability in colour pattern may be a typical feature of the entire genus.

The apparent rarity that characterises all species of the genus *Callophylla* is probably related to their unusual life cycle and ecology. Unfortunately, little information is yet available on the habitat and feeding habits of adults. One specimen of *C.costata* was reportedly collected inside a termite nest in southern Cameroon (Thierry Garnier, pers. comm.), while the label accompanying the only known specimen of *C.takanoi* explicitly states that it was collected in a “yellow pan trap” at an altitude of 1400 m (Legrand 2015). There is no evidence, however, to suggest that adults may feed on either flowers, fruits or sap flows. These and other details (e.g., “Nakonde Hochland”, “Mont Kala”, “Mont Nimba”), also seem to point towards a mountainous habitat for the genus.

Concerning period of adult activity, the scarce records available in the literature and collections in general indicate that adults of this genus may only be active for short periods, possibly after major rainfall events. Collection records range from March to December, with most in March/April (Sakai and Nagai 1998, Antoine 2007, Legrand 2015, Thierry Garnier and Gerhard Beinhundner pers. comm.).

## Supplementary Material

XML Treatment for Callophylla macrocephala
